# Senescent renal tubular cells derived extracellular vesicles transported miR-20a and miR-21 induced macrophage-to-myofibroblast transition in renal fibrosis after ischemia reperfusion injury

**DOI:** 10.7150/ijbs.97579

**Published:** 2025-01-06

**Authors:** Qiang Zhong, Jun Zeng, Yue Li, Haohan Zhang, Tao Lin, Turun Song

**Affiliations:** 1 Department of Organ Transplantation Center, Affiliated Hospital of Zunyi Medical University, Zunyi, Guizhou, China.; 2 Department of Urology, Institute of Urology, Organ Transplantation Center, West China Hospital, Sichuan University, Chengdu, Sichuan, China.

**Keywords:** cell senescence, macrophage-myofibroblast transition, extracellular vesicles, microRNA, renal fibrosis, ischemia-reperfusion injury

## Abstract

In our investigation, we aimed to shed light on the role of senescent cells in renal fibrosis, considering the observed correlation between renal tubular epithelial cell senescence and the presence of renal fibrosis. Our findings confirm the manifestation of senescence characteristics in renal tubular epithelial cells during renal fibrosis and establish their capacity to trigger a transition from macrophages to myofibroblasts, known as macrophage-myofibroblast transition (MMT). Additionally, our study uncovered that extracellular vesicles released by senescent HK-2 cells (sHK-2) play a pivotal role in facilitating MMT. Subsequently, we investigated the miRNA profile in sHK-2-derived extracellular vesicles (sHK-2-EVs) and confirmed the elevated abundance of specific miRNAs, including miR-20a-5p and miR-21-5p, compared to normal HK-2-EVs. Notably, these miRNAs possess the capability to induce M2-like polarization in macrophages and enhance the expression of TGF-β. Moreover, TGF-β can stimulate macrophages to produce miR-20a-5p and miR-21-5p, establishing a positive feedback loop that amplifies the TGF-β/Smad pathway and facilitates the process of macrophage-myofibroblast transition.

## Introduction

In recent years, numerous studies have emerged highlighting the dual role of renal tubular epithelial cells (TECs) in acute kidney injury (AKI). These investigations have revealed that TECs not only experience damage during AKI but also contribute significantly to the pro-inflammatory and pro-fibrotic processes within renal tissue. Additionally, these findings suggest that TECs may play a pivotal role in the transition from AKI to chronic kidney disease (CKD)^1^, ^2^. Impaired TECs exhibit distinct characteristics such as cell cycle arrest, elevated levels of senescent biomarkers (p21 and p16), heightened SA-β-Gal activity, and increased secretion of pro-inflammatory and pro-fibrotic factors^3^, ^4^. Consequently, it is apparent that impaired renal tubular cells undergo cell senescence and display a senescence-associated secretory phenotype (SASP), thereby potentially contributing to inadequate repair and the progression of renal fibrosis following AKI^3^, ^4^. Conversely, in animal models of renal fibrosis, the administration of drugs aimed at clearing senescent cells has demonstrated significant reductions in the extent of renal fibrosis^5^-^7^.

Renal fibrosis is characterized by the excessive accumulation of extracellular matrix in the renal interstitium, predominantly contributed by myofibroblasts^8^. Previous research has indicated various sources of myofibroblasts, including fibroblast activation, epithelial transition, endothelial transition, pericyte transition, and macrophage transition (known as macrophage to myofibroblast transition, MMT)^9^, ^10^. Notably, studies have reported that approximately 50% of myofibroblasts found in renal graft biopsy tissues with chronic active rejection originate from bone marrow-derived macrophages^11^. Furthermore, selective removal of macrophages has shown promising results in alleviating renal fibrosis in animal models of fibrosis^12^. Retrospective analysis of renal allograft biopsies has also revealed the frequent presence of CD163+ macrophages in regions of interstitial fibrosis, characterized by the accumulation of type I collagen and the clustering of myofibroblasts expressing α-smooth muscle actin (α-SMA)^13^. Additionally, the quantity of CD163+ macrophages during the early and later years correlates with the degree of interstitial fibrosis observed in the fifth- and tenth-years post-transplantation, respectively^13^. To summarize, the significant involvement of macrophages and MMT in the progression of renal fibrosis has been established; however, the precise mechanism underlying MMT remains elusive.

Extracellular vesicles(EVs) are cellular structures enclosed by a lipid bilayer and contain a diverse array of proteins, lipids, and nucleic acids, and they are actively secreted by cells^14^. These vesicles have emerged as key players in facilitating communication between senescent cells and neighboring cells within the microenvironment. Furthermore, extracellular vesicles serve as vehicles for the transport of various miRNAs, which cells selectively release into the surrounding environment to target cells in response to different stimuli^15^. Prior research has highlighted the involvement of specific microRNAs, such as the miR-196 family, miR-200 family, miR-21, and miR-192, in renal fibrosis, where they have been shown to contribute to its progression^16^. While the roles of senescent renal TECs and MMT in renal interstitial fibrosis have been established, their precise relationship remains uncertain. Therefore, we hypothesize that EVs secreted by senescent renal tubular epithelial cells may interact with macrophages and induce MMT, thereby contributing to the progression of renal fibrosis. The microRNAs encapsulated within these vesicles may play a crucial role as key mediators. This study presents groundbreaking findings demonstrating that vesicles released by senescent renal tubular cells can induce MMT. Additionally, the study reveals that the miR-20a/miR-21-TGF-β axis can drive macrophages towards M2-like polarization and promote the process of MMT.

## Methods

### Human renal allograft biopsy samples

Renal biopsy samples were procured from the biological specimen bank at West China Hospital in Chengdu, China. The control group samples were collected promptly following transplantation, while the experimental group samples were obtained from patients diagnosed with chronic rejection based on the Banff 2019 criteria. All the recipients received live donor kidneys. Kidney transplant procedures were conducted within the time frame spanning from 2014 to 2018. None of the organs were procured from executed prisoners and that organs were procured after informed consent. The study received approval from the Ethics Committee of West China Hospital (Approval Number: 2019748).

### Animals

Male wild-type C57BL/6J mice, aged 6-8 weeks, were obtained from DOSSY Experimental Animals Co. Ltd. (Chengdu, China). The mice were housed in a specific pathogen-free (SPF) room with a regulated 12-hour light/dark cycle, maintaining a constant temperature (23-24°C) and humidity (55 ± 5%). Throughout the study, the mice had ad libitum access to food and water. All animal experiments were conducted following the guidelines and regulations set by the animal ethics committee of the University of Sichuan (Approval Number: 20220120001).

### Kidney IR model and experimental protocol

Anesthesia was induced with a high dose of isoflurane inhalation (4%) and maintained with a lower dose (2%) using a vaporizer on a warming pad at 37°C. An abdominal incision was made, and vascular clamps were applied to both renal pedicles for 35 minutes(bilateral IRI). The kidneys were re-perfused, and the incision was sutured for recovery. The same procedure was followed for the control group, without occlusion of the renal arteries. Mice were sacrificed on postoperative day 2 and day 28. In the dasatinib+quercetin (D+Q) treatment group, dasatinib (5mg/kg, based on body weight) and quercetin (50mg/kg) were administered via oral gavage in 100-150μL of 10% PEG400 solution^17^. The D+Q treatment was given on postoperative days 1, 2, 3, and 8, 9, 10. Dasatinib and Quercetin were obtained from MedChemExpress (MCE, Shanghai, China).

### Renal function and histology

The levels of serum creatinine in the mice were assessed using a mouse Creatinine Assay Kit employing the Sarcosine Oxidase method. Additionally, blood urea nitrogen (BUN) levels were measured using a Urea Nitrogen Kit based on the Urease method. To examine kidney histology, formalin-fixed sections were prepared and stained with H&E (G1120, Solarbio) as well as Masson's trichrome (G1340m, Solarbio).

### Cell culture and treatment

The HK-2 cells (Procell, CL-0109) and THP-1 cells (Procell, CL-0233) were initially obtained from Procell Life Science and Technology Co., Ltd. (Wuhan, China). HK-2 cells were cultured in DMEM with 10% FBS and 1% penicillin/streptomycin, while THP-1 cells were grown in RPMI-1640 with 10% FBS, 1% penicillin/streptomycin, and 0.05 mM β-mercaptoethanol. Paclitaxel was added to HK-2 cells at concentrations ranging from 10 nM to 100 μM in medium with 10% FCS^5^. After 24 hours of PTX treatment, the drug was removed, and cells were cultured for an additional 24 hours to collect supernatant samples or co-cultured with THP-1-derived macrophages for 48 hours. THP-1 cells were treated with 100 ng/ml PMA (Phorbol 12-myristate 13-acetate, Cat# HY-18739, MCE, China) for 24 hours to induce adherence and obtain macrophages.

### EVs isolation

Following a 24-hour treatment of HK-2 cells with paclitaxel (PTX), the drug was removed by washing the cells, which were then cultured in DMEM medium supplemented with 10% EVs-depleted FBS (SBI, American) for an additional 24 hours. Subsequently, the cell supernatant obtained from this culture was utilized for the isolation of EVs through a process of differential centrifugation (More details in Supplementary Methods).

### H&E, Masson's trichrome and Immunohistochemical (IHC) staining

For H&E and Masson's trichrome staining, paraffin-embedded sections of mouse kidney and human renal biopsy samples were utilized following standard procedures. The stained sections were examined using a light microscope (Olympus, Tokyo, Japan). Immunohistochemistry (IHC) staining was conducted using the G1215 IHC kit (ServiceBio). Kidney tissues were fixed, embedded, and sectioned before being incubated with primary antibodies overnight at 4°C. After washing, the sections were treated with HRP-conjugated IgG, developed with DAB, and counter-stained with hematoxylin.

### Immunofluorescence staining

Macrophages were treated and cultured on glass coverslips in six-well plates. The cells were fixed, permeabilized, and blocked before being incubated with primary antibodies overnight at 4°C. The tyramide signal amplification (TSA) system with Opal dye reagents was used. The multiplexed slides were observed using a confocal microscope (Nikon C2, Tokyo, Japan).

### *In vitro* microRNA transfection

Ribobio Biotech (Guangzhou, China) synthesized all the miRNA mimics (miR-20a-5p, miR-21-5p), inhibitors (miR-20a-5p, miR-21-5p), and their corresponding perturbation controls (miR-NC) utilized in this study. MicroRNA transfection was carried out using riboFECTTM CP transfection reagent (C10502) from RiboBio (Guangzhou, China) following the manufacturer's instructions.

### Real-time quantitative polymerase chain reaction (RT-qPCR)

Total RNA was extracted using the FastPure Cell/Tissue Total RNA Isolation Mini Kit (Vazyme, Nanjing, China), and cDNA synthesis was performed using the HiScript II One-Step RT-PCR Kit (Vazyme, Nanjing, China). Quantitative RT-PCR analysis was conducted using the AceQ Universal SYBR qPCR Master Mix (Vazyme, Nanjing, China) on a CFX-96 PCR Real-Time PCR Detection System (Bio-Rad, CA, USA). GAPDH served as the internal control for mRNA quantification. For miRNA analysis, reverse transcription was carried out using the microRNA Reverse Transcription Kit (Tiangen Biotech, Beijing, China), and the miRNA levels were normalized to the U6 small nuclear RNA as an endogenous control. The relative expression of both mRNA and miRNA was determined using the 2^-∆∆CT method. Please refer to **Supplementary Table 1** for the primer sequences used for miRNA and mRNA quantification.

### Western blot analysis

Cells were washed with PBS, then lysed with RIPA buffer containing protease and phosphatase inhibitors (Biosharp, Hefei, China). The protein concentration was determined using the BCA protein assay reagent, and 20-30 µg of total protein samples were separated on 4-12% or 4-20% SurePAGETM Bis-Tris precast gels (Genscript, Nanjing, China) under a constant voltage of 120V. Proteins were transferred onto PVDF membranes (Millipore, USA). The membranes were blocked with milk, incubated with primary antibodies overnight at 4°C, washed, and then incubated with secondary antibodies. Protein was visualized and quantified using ImageJ software (version 2.0.0, ImageJ, Marlyand, USA).

### Statistical analysis

The data are presented as the mean ± standard deviation (SD). Statistical analyses were performed using GraphPad Prism 8 software (GraphPad Software Inc., San Diego, USA). The data were obtained from results of at least three independent experiments. A t test was utilized to analyze the differences between groups and a one-way analysis of variance (ANOVA) test with Tukey's post hoc test was utilized for assessing the differences of mean values of more than two groups. A p-value less than 0.05 was considered statistically significant.

Further methods are available in the **Supplementary Methods**.

## Results

### Identification of senescence TECs and MMT in human chronic renal allograft injury and mouse model of renal fibrosis

**Figure 1:**
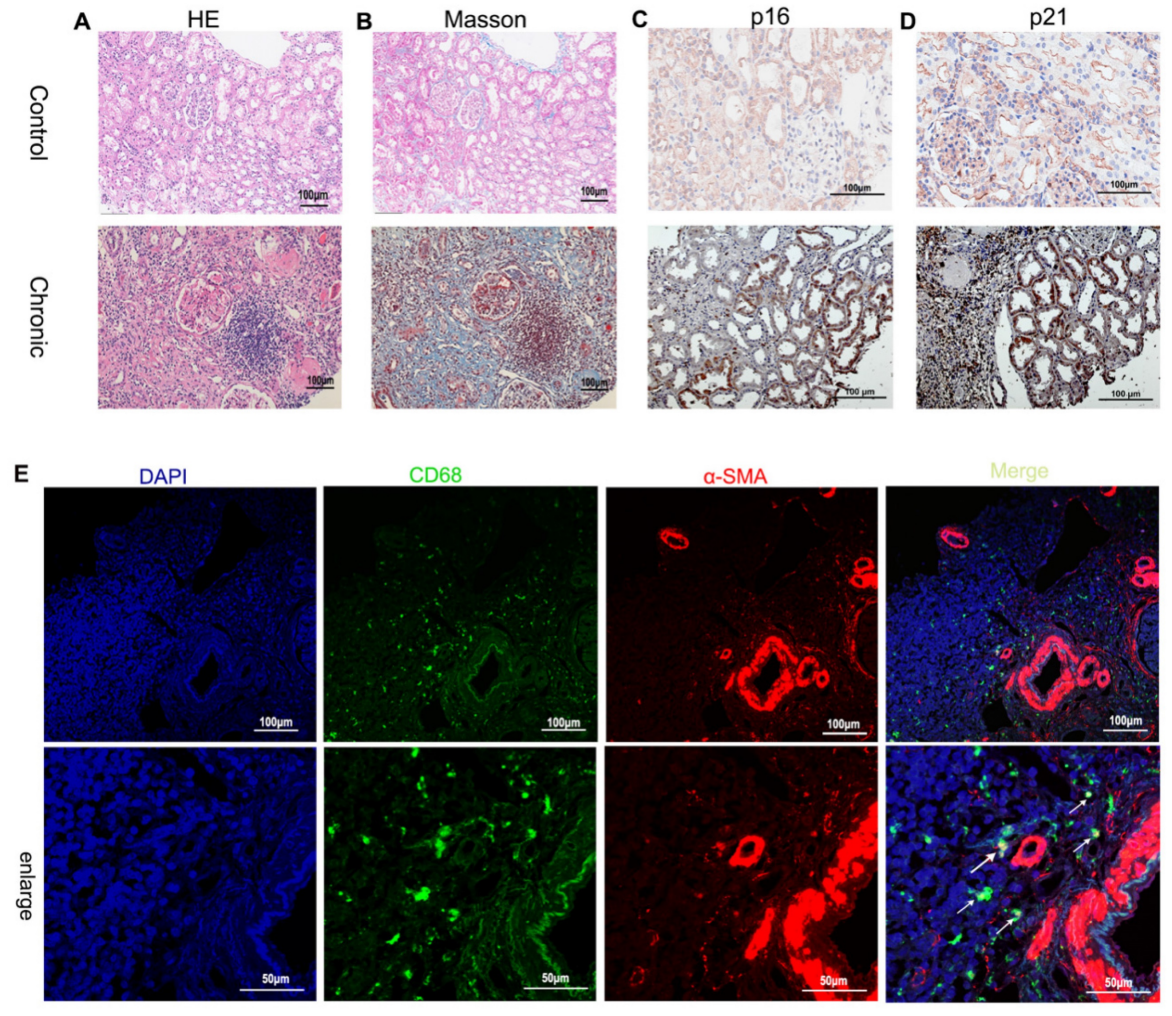
** Renal fibrosis, senescence of renal tubular epithelial cells and MMT in patients with chronic active renal transplantation rejection.** (A) H&E staining. (B) Masson trichrome staining (blue). (C, D) Immunohistochemical staining of p16 and p21 renal biopsy tissue revealed the senescence of renal tubular epithelial cells. (E) MMT in patients with chronic active renal transplantation rejection. Twocolor immunofluorescence identifies MMT cells that co-express macrophage (CD68, green) and myofibroblast (α-SMA, red) markers in biopsy tissues. Arrows indicate CD68- and α-SMA-positive cells (MMT cells).

To investigate the presence of renal tubular cell senescence during the progression of renal fibrosis, immunohistochemical staining was performed on biopsy samples from patients with chronic renal graft rejection (**Supplementary Table 2**) to assess the expression of p21 and p16, known markers of cellular senescence. The results revealed a significant presence of p21- and p16-positive TECs in the microscopic field, indicating the association between chronic graft fibrosis and TEC senescence (**Figure 1 A~D**). Immunofluorescence staining was further employed to identify cells undergoing MMT based on the co-expression of macrophage (CD68) and myofibroblast (α-SMA) markers. The biopsy specimens from patients with chronic active renal graft rejection exhibited the presence of CD68+ macrophages and α-SMA+ cells, with numerous double-labeled positive cells (**Figure 1 E**).

**Figure 2:**
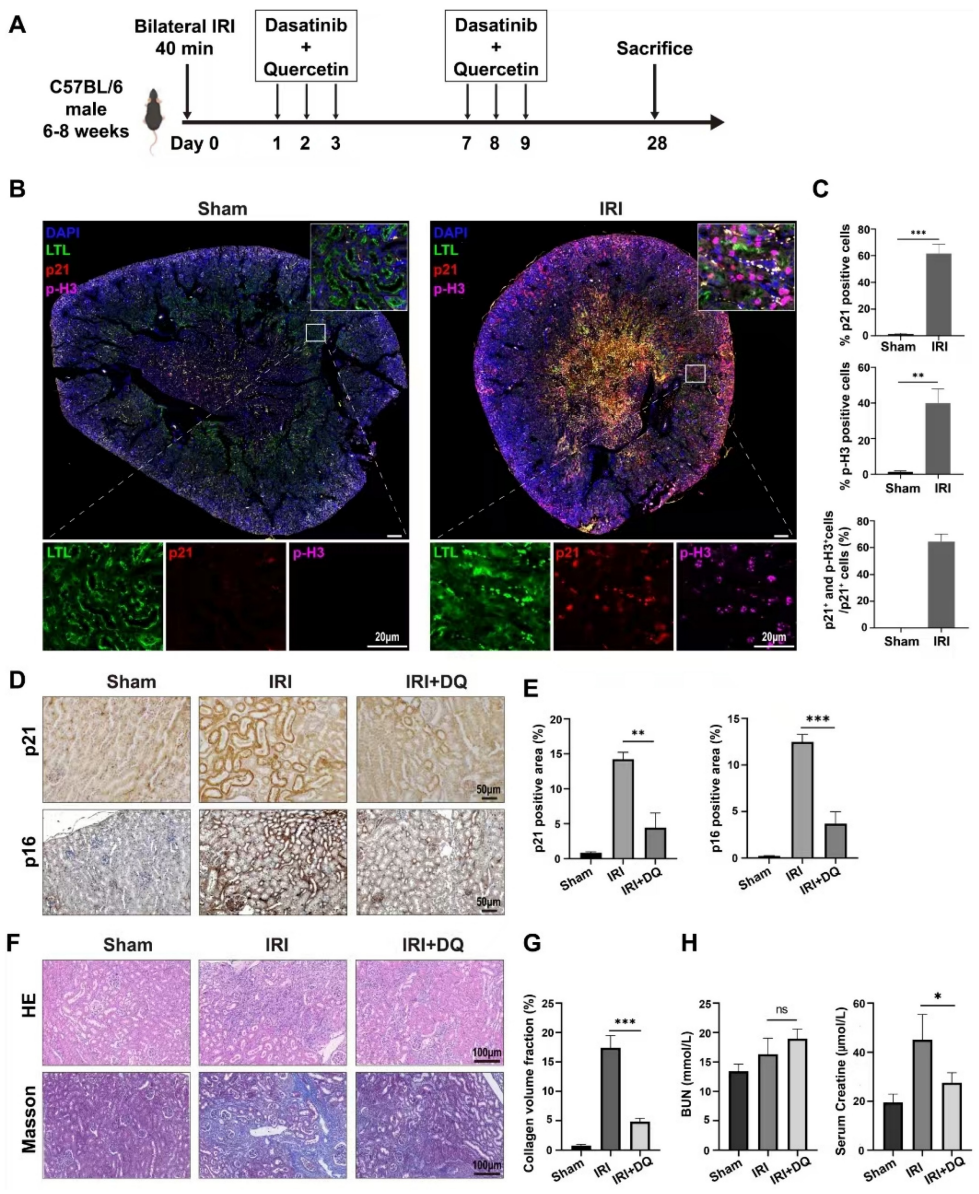
** Senescence of renal tubular epithelial cells and the role of senescent cells in the progression of renal fibrosis after ischemia-reperfusion injury in mice.** (A) Schematic presentation of the main experimental protocol. (B) Multiplex-immunofluorescence staining images showed the presence of the senescent tubular cells and infiltration of F4/80+ macrophage. p16- and p-H3(ser10)-positive cellsrepresent senescent renal tubular cells with cell cycle arrest at G2/M phase. (C) Quantification of the number of p16+ cells, p-H3(ser10)+ cells, and p16+p-H3(ser10)+ cells as a percentage of total p21+ cells (cell counts per field) . Data are mean±SD for groups of 4 mice. (D, E) Immunohistochemical staining for p21 and p16. Graphs show quantification of the area of p21 and p16 staining.Data are mean±SD for each group of 6 mice. (F, G) Immunohistochemical staining for Masson trichrome stain. Graphs show quantification of the area of collagen deposition using Masson trichrome staining. Data are mean±SD for each group of 6 mice. (H) Blood biochemical values of blood urea nitrogen and creatinine at day 28 post-operation.***P<0.001; **P<0.01; *P<0.05; ns, not significant.

**Figure 3:**
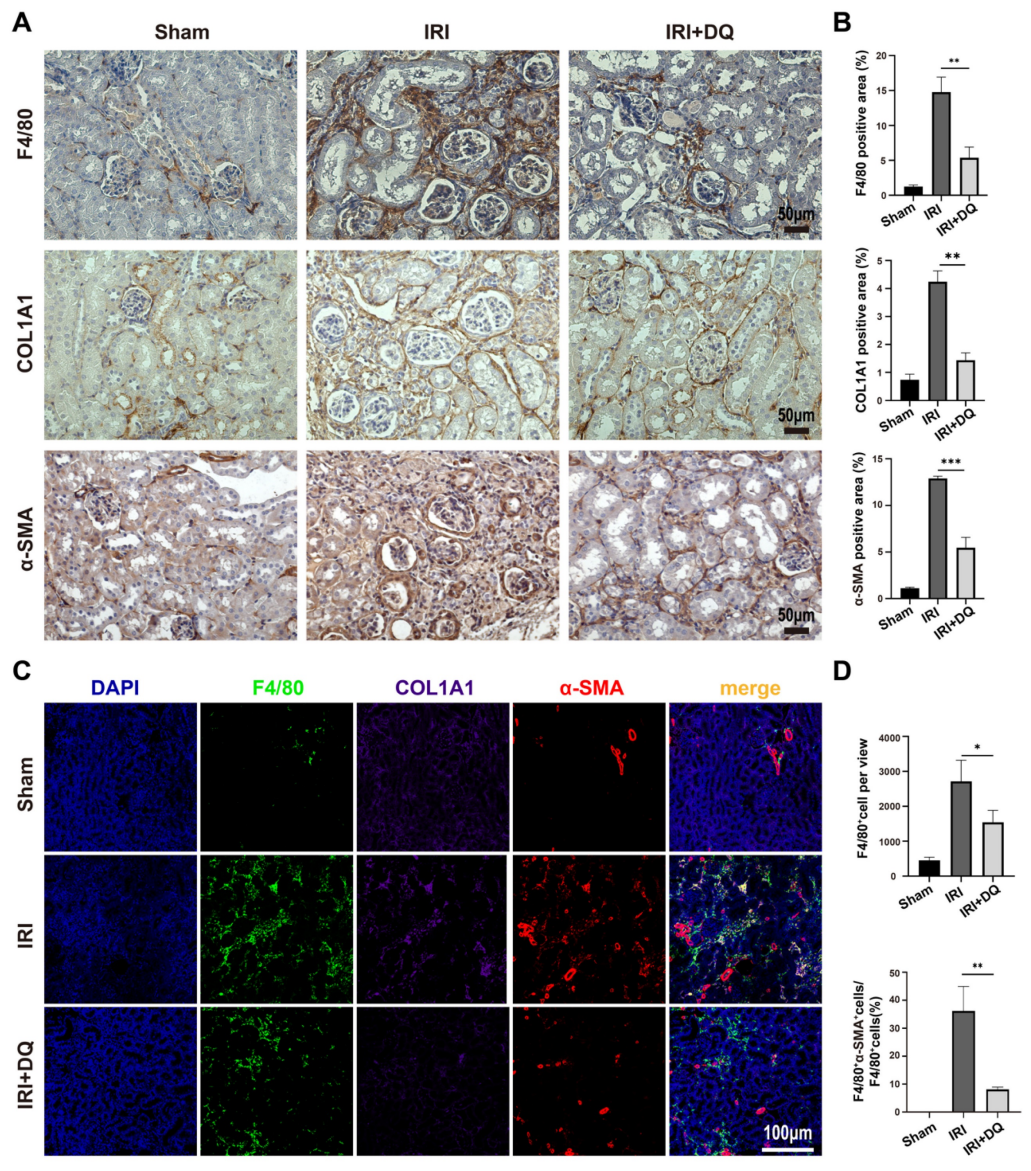
**The number of infiltrating macrophages and MMT cells decreased after senescent tubular cells were removed.** (A, B) Immunohistochemical staining for F4/80, COL1A1 and α-SMA. Graphs show quantification of the area of F4/80, COL1A1 and α-SMA staining. (Data are mean±SD for groups of 4 mice). (C) Multiplex-immunofluorescence staining images showed the infiltration of F4/80+ macrophage and the MMT cells(F4/80+α-SMA+cells) in renal interstitial tissue (yellow regions). (D) Quantification of the number of F4/80+ macrophage, and F4/80+α-SMA+ cells as a percentage of total F4/80+ cells (cell counts per field).Data are mean±SEM for each groups of 4 mice.**P<0.01; *P<0.05.

Next, a IRI model (bilateral IRI, 37℃, 35min) was utilized in mice to investigate fibrosis. Immunofluorescence staining was conducted on kidney specimens taken on the second day after surgery. The findings indicated a considerable number of p21-positive cells were observed in the renal tubules, along with a substantial proportion of TECs expressing p-H3, a marker indicating G2/M phase (**Figure 2 B**). These differences were statistically significant (P<0.01) (**Figure 2 C**). Confocal microscopy revealed the coexistence of p-H3 and p21 double-positive tubular cells, suggesting that senescent TECs in the IRI group were arrested at the G2/M phase, constituting 63.4%±5.6% of the total p21-positive TECs (**Figure 2 B, C**). Immunohistochemical staining and multiplex immunofluorescence staining were performed as planned on kidney specimens obtained 28 days after IRI (**Figure 2 A**). The findings demonstrated a significantly higher extent of interstitial fibrosis, and renal tubular cells expressing p21 and p16 in the IRI group compared to the sham-operated group (p<0.01) (**Figure 2 D~H**). Three-color confocal microscopy revealed numerous F4/80+ α-SMA+ MMT cells in the renal interstitium surrounded by deposited collagen I, whereas the sham-operated group displayed no double-positive cells (**Figure 3 C**).

To assess the impact of clearing senescent TECs on renal fibrosis and MMT, dasatinib and quercetin (DQ) were administered orally on postoperative days 1, 2, 3 and 7, 8, 9 (**Figure 2 A**). Effective clearance of senescent TECs in the IRI+DQ group led to a significant reduction in renal fibrosis compared to the IRI group (**Figure 2 F,G**). Furthermore, the infiltration of F4/80+ macrophages was significantly decreased (P<0.05) (**Figure 3 A~D**), along with a substantial reduction in the number of F4/80+α-SMA+ MMT cells (P<0.01) (**Figure 3 C, D**). These findings suggest that senescent tubular cells may play a crucial role in macrophage infiltration and the transition of macrophages to myofibroblasts during renal fibrosis.

### Senescent renal tubular epithelial cells promoted MMT

**Figure 4:**
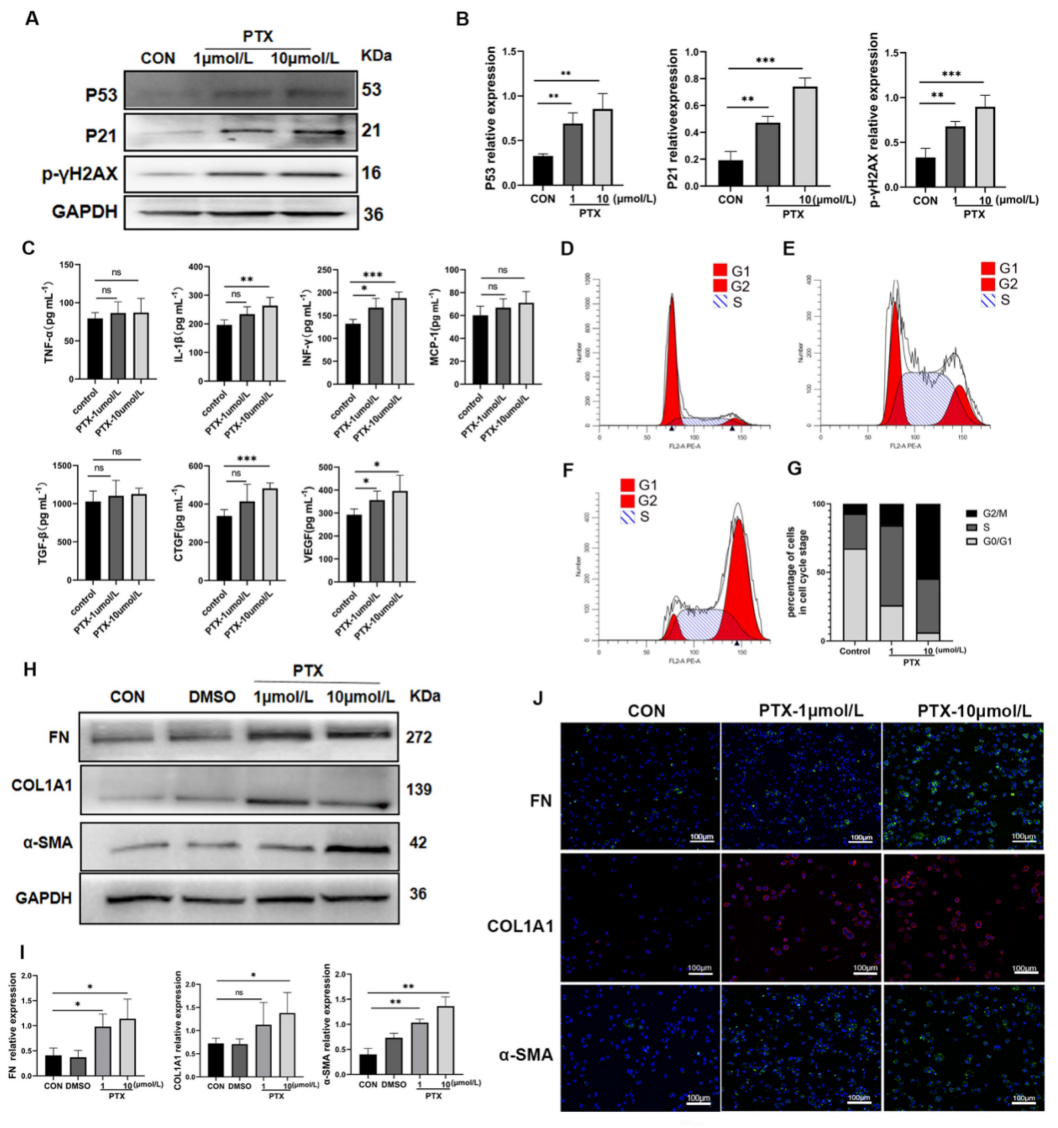
**Senescent HK-2 cells induce MMT.** (A) Western bolt analysis reveals the protein expression of p53, p21, p16, γH2AX (senescence markers) were elevated in HK-2 cells treated with paclitaxel (PTX) (1μmol/L and 10μmol/L) for 24 hours. (B) Quantified data of immunoblotting band intensity in (A). (C) The concentrations of TNF-α, IL-1β, INF-γ, MCP-1, TGF-β, CTGF and VEGF in supernatants from senescent HK-2 cells were all elevated, but the differences between TNF-α, MCP-1 and TGF-β and the control group were not statistically significant. (D, E, F) Cell cycle analysis by propidium iodide staining and flow cytometry in HK-2 cells treated with paclitaxel at various doses for 24h. (G) Cell cycle distribution of HK-2 cells treated with paclitaxel at various doses for 24h. (H) Western bolt analysis reveals the protein expression of FN, COL1A1, α-SMA were elevated in THP-1-drived macrophage co-cultured with HK-2 cells seeded on top of the transwell system for 48h. (I) Quantified data of immunoblotting band intensity in (H). (J) Immunofluorescence images confirm substantial expression of FN, COL1A1, α-SMA in THP-1-drived macrophage. ***P<0.001; **P<0.01; *P<0.05; ns, not significant.

To investigate the potential of senescent TECs in inducing MMT, we conducted transwell co-culture experiments using senescent HK-2 cells and THP-1 cell-derived macrophages *in vitro*. Based on previous studies^18^ and our own animal experiments that highlighted the importance of TECs arresting at the G2/M phase for renal interstitial fibrosis, we utilized different concentrations of paclitaxel (PTX) (1μmol/L and 10μmol/L) to induce senescence in HK-2 cells. We assessed senescence markers, including p53, p21, p16, γH2AX protein expression, senescence-associated β-galactosidase (SA-β-Gal) activity, cell cycle, and SASP (**Figure 4 A~G, Supplementary Figure 1**). Notably, the levels of IL-1β, INF-γ, TGF-β, CTGF, and VEGF in the supernatants of senescent HK-2 cells were significantly increased (P<0.05). Conversely, the differences in the concentrations of TNF-α, MCP-1, and TGF-β between the senescent group and the control group were not statistically significant (**Figure 4C**). In the transwell co-culture experiments, macrophages co-cultured with senescent HK-2 cells treated with 10μmol/L PTX exhibited significantly higher protein expression of fibrotic markers (including FN, COL1A1, and α-SMA) compared to the control group, with statistically significant differences (**Figure 4 H, I**). The findings from immunofluorescent cytochemistry staining were consistent with the results obtained from Western blot analysis (**Figure 4 J**).

### Senescent renal tubular cell derived EVs promoted MMT

**Figure 5:**
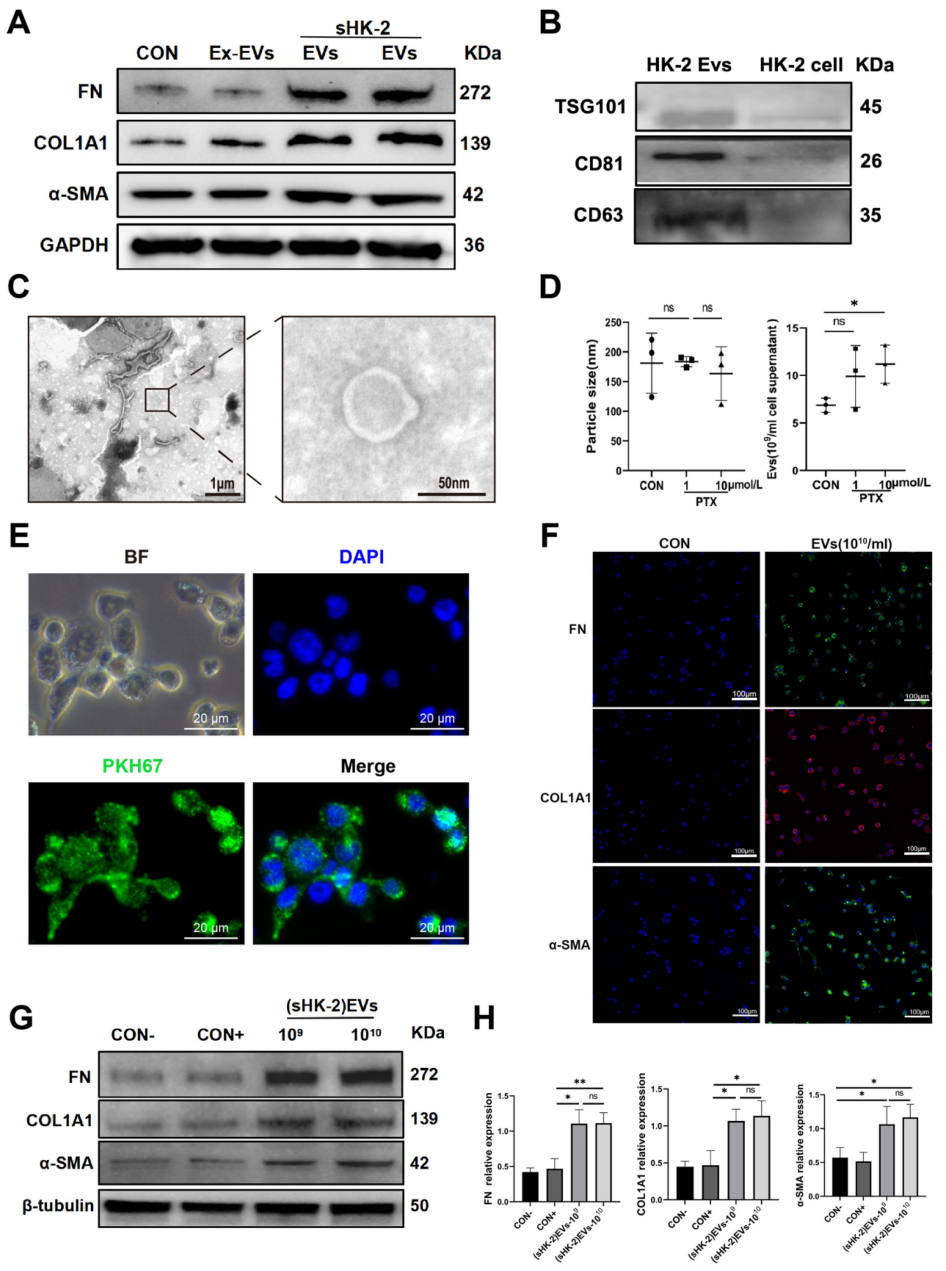
** Senescent HK-2-derived EVs promoted MMT.** (A) Western bolt analysis reveals the protein expression of FN, COL1A1, α-SMA were elevated in macrophage co-cultured with senescent HK-2-derived EVs compare with EVs-excluded (Ex-EVs) supernatants or normal HK-2-drived EVs. (B) Protein immunoblots of EVs, including three typical exosomal markers (TSG101, CD63 and CD81). (C) A representative TEM image of EVs. (D) Particle size distribution and quantification of EVs was measured using nanoparticle tracking analysis (NTA). (E) Immunofluorescence images reveal the uptake of sHK-2-EVs by macrophage after co-culture with PKH67-labelled EVs for 24 h. (F) Western blot analysis of FN, COL1A1, α-SMA were elevated in macrophage co-cultured with sHK-2 EVs at various concentrations compare with negative control(con-) or normal HK-2-drived EVs(con+). (G) Quantified data of immunoblotting band intensity in (F). (H) Immunofluorescence images confirm substantial expression of FN, COL1A1, α-SMA in macrophage. **P<0.01; *P<0.05; ns, not significant.

Extracellular vesicles (EVs), characterized by their lipid bilayer structure, have the ability to carry specific lipids, proteins, and nucleic acids for communication with the external environment and neighboring cells^19^. Based on this, we hypothesized that EVs derived from senescent renal tubular cells could induce MMT. To investigate this hypothesis, we first isolated EVs from the supernatant of senescent HK-2 cells and normal HK-2 cells using differential centrifugation. The EVs were then characterized by examining the expression of TSG101, CD63, and CD81 proteins, and their typical morphology was confirmed using TEM and NTA; A concentration of extracellular vesicles ranging from 10^9^ to 10^10^/ml was obtained, with a particle size between 112-220nm. (**Figure 5 B, C, D**). Subsequently, we labeled the EVs with PKH67 dye and co-cultured them with macrophages, demonstrating that the EVs were effectively taken up by the macrophages (**Figure 5 E**).

For our control, we used supernatants devoid of EVs (Ex-EVs) post-centrifugation for co-culture with macrophages. Our findings showed that EVs from senescent HK-2 cells markedly upregulated FN, COL1A1, and α-SMA protein levels compared to the Ex-EVs control (**Figure 5A**). Additionally, the CON+ group, acting as a positive control and consisting of EVs from normal (6-8 passages) HK-2 cells, did not influence the expression of these proteins in macrophages, as depicted in **Figure 5F**. Therefore, combined with the results shown in **Figure 5A**, it validates that EVs derived from senescent HK-2 cells, rather than those from normal HK-2 cells, are responsible for the elevated levels of α-SMA, FN, and COL1A1 proteins within the macrophages (**Figure 5 F, G**). The findings from immunofluorescent cytochemistry staining were consistent with the results obtained from Western blot analysis (**Figure 5 H**).

### MiR-20a-5p and miR-21-5p in sHK-2-EVs promoted MMT

**Figure 6:**
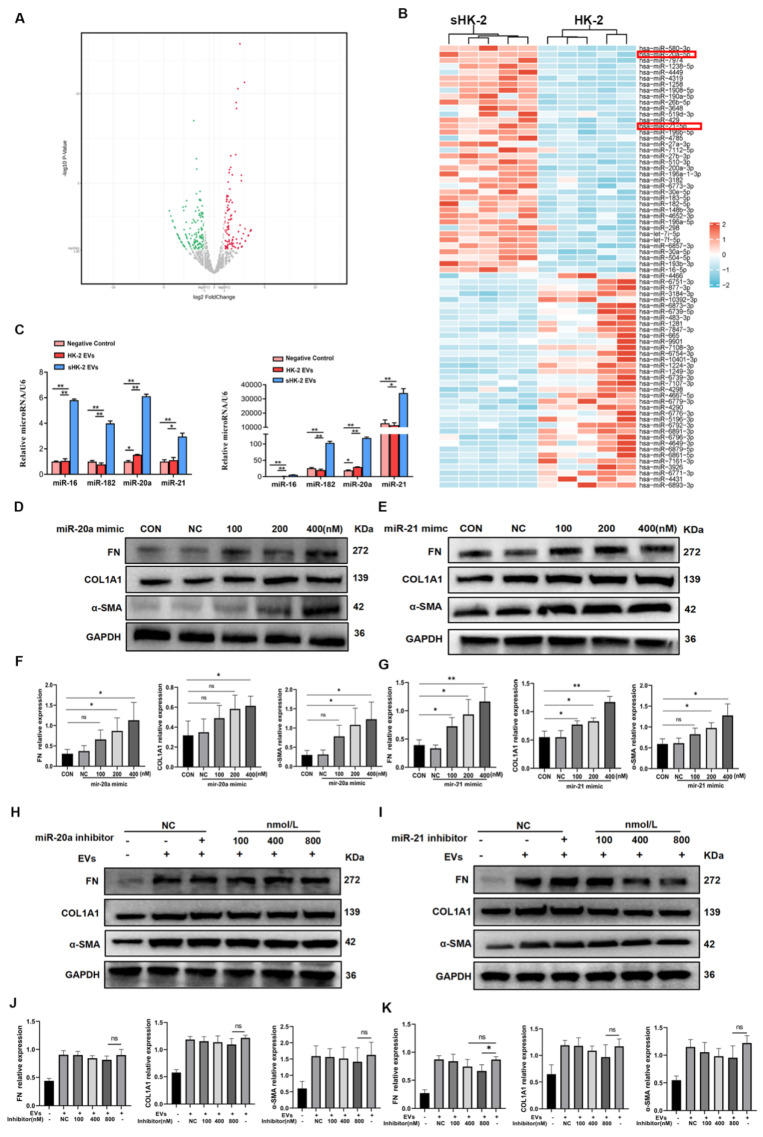
**MiR-20a-5p and miR-21-5p in sHK-2-EVs promoted MMT.** (A) A volcano plot shows differentially expressed miRNAs with statistical significance and fold change in the normal HK-2 EVs and the sHK-2 EVs group. (B) Heat map showing the differential expressed miRNAs between normal HK-2 EVs and the sHK-2 EVs group. (C) The significantly high-expressed miRNAs in sHK-2 EVs were verified by qPCR. Compared with the respective negative control group, the fold changes of miRNAs expression after adding EVs(left); compared with the miR-16 negative control group, the fold changes of miRNAs expression after adding EVs(right). (D) Macrophage were transfected with miR-20a-5p mimic or control miR for 48 hours, after which FN, COL1A1, α-SMA protein expression was assayed by western blot. (E) Macrophage were transfected with miR-21-5p mimic or control miR for 48 hours, after which FN, COL1A1, α-SMA protein expression was assayed by western blot. (F) Quantified data of immunoblotting band intensity in (D). (G) Quantified data of immunoblotting band intensity in (E). (H) Macrophage were transfected with miR-20-5p inhibitor or control miR, and co-cultured with sHK-2 EVs (10^10^/ml) for 48 hours, after which FN, COL1A1, α-SMA protein expression was assayed by western blot. (I) Macrophage were transfected with miR-21-5p inhibitor or control miR, and co-cultured with sHK-2 EVs (10^10^/ml) for 48 hours, after which FN, COL1A1, α-SMA protein expression was assayed by western blot.(J) Quantified data of immunoblotting band intensity in (H). (K) Quantified data of immunoblotting band intensity in (I). **P<0.01; *P<0.05; ns, not significant.

Previous studies have highlighted the enrichment of miRNAs in EVs, with cells actively and selectively releasing miRNAs into the surrounding environment in response to various stimuli^15^. In line with this, we conducted RNA-seq analysis of EVs derived from normal and senescent HK-2 cells to identify potential miRNA effectors. Differential expression analysis revealed several miRNAs with distinct expression patterns, and a heat map was generated to display the top 75 miRNAs exhibiting the most significant differences between the two groups (**Figure 6 A, B**). Considering the relative abundance of miRNAs in senescent HK-2 cells, we focused on miR-20a-5p and miR-21-5p as candidate miRNAs for further investigation.

To validate the reliability of the sequencing results, we performed qPCR validation of four miRNAs (miR-20a-5p, miR-21-5p, miR-182-5p, miR-16-5p) in macrophages co-cultured with sHK-2-EVs. Notably, miR-20a-5p and miR-21-5p exhibited significantly higher expression levels in macrophages cultured with sHK-2-EVs compared to those cultured with normal HK-2-EVs (**Figure 6 C**). Subsequently, we confirmed the efficacy of miR-20a and miR-21 mimics transfection into macrophages (**Supplementary Figure 2**) and proceeded to assess the impact of miR-20a-5p and miR-21-5p mimics and inhibitors on macrophage protein expression. Increasing concentrations of miR-20a and miR-21 mimics correlated with a gradual elevation in the protein levels of FN, COL1A1, and α-SMA in macrophages (**Figure 6 D~G**). Conversely, escalating concentrations of miR-20a and miR-21 inhibitors led to a gradual reduction in the expression of fibrosis markers FN, COL1A1, and α-SMA. Notably, only the high concentration of miR-21 inhibitor (800nM) significantly counteracted the promoting effect of sHK-2-EVs on FN expression in macrophages (**Figure 6 H~K**).

### MiR-20a-5p and miR-21-5p promoted macrophage M2 polarization and formed a positive feedback loop with TGF-β

To investigate the impact of miR-20a-5p and miR-21-5p on macrophage polarization, we conducted western blot and qPCR assays on macrophages co-cultured with these two miRNA mimics. The results demonstrated a gradual increase in both mRNA and protein expression of CD163 in macrophages as the concentration of added mimics increased, exhibiting statistically significant differences compared to the control group (**Supplementary Figure 3 A~E**). We further assessed the concentration of TGF-β1 in the supernatant following co-culture of these miRNA mimics with macrophages and observed that higher concentrations of miR-20a and miR-21 mimics corresponded to significantly elevated levels of TGF-β in the supernatant, showing statistically significant differences compared to the control group (**Supplementary Figure 3 F, G**). Moreover, to investigate whether TGF-β could induce the production of miR-20a-5p and miR-21-5p in macrophages, we added TGF-β1 at concentrations of 5ng/ml and 10ng/ml to the macrophage medium and analyzed the levels of these two miRNAs using qPCR. The results revealed significantly higher levels of miR-20a-5p and miR-21-5p in macrophages compared to the control group (**Supplementary Figure 3 H, I**). Collectively, these findings suggest the existence of a positive feedback loop between miR-20a-5p/miR-21-5p and TGF-β, where they can activate each other's production.

### MiR-20a-5p and miR-21-5p inhibited Smad7 expression and promoted Smad3 phosphorylation

To investigate the regulatory mechanisms of miR-20a-5p and miR-21-5p on MMT, we utilized three biological prediction software programs, namely TargetScan, miRDB, and miRTarBase, to identify potential target genes regulated by these miRNAs (**Supplementary Figure 4 A**). Subsequently, KEGG analysis was conducted to identify genes enriched in the TGF-β/Smad pathway (**Supplementary Figure 4 B, C, D**). Among the candidate genes, Smad7 was of particular interest due to its known negative regulatory effect on the TGF-β/Smad signaling pathway. TargetScan software predicted potential direct binding sites for both miR-20a and miR-21 in the 3'UTR of smad7 mRNA (**Supplementary Figure 5 A**). To validate this prediction, a dual luciferase reporter assay was performed (**Supplementary Figure 5 B, C**). Subsequently, we assessed the protein expression levels of Smad7, Smad3, and phosphorylated Smad3 in macrophages following co-culture with miR-20a-5p and miR-21-5p mimics and inhibitors. The results revealed that increasing concentrations of miRNA mimics led to a decrease in Smad7 protein expression and a significant increase in phosphorylated Smad3 levels (**Supplementary Figure 5 D~J**). These findings suggest that miR-20a-5p and miR-21-5p can inhibit Smad7 expression, thereby promoting the activation of the TGF-β signaling pathway.

## Discussion

This study aimed to investigate the regulatory mechanisms underlying senescent renal tubular epithelial cells and their role in the process of MMT. Our findings demonstrated that following renal I/R injury, a subset of renal TECs exhibited characteristics of cellular senescence and were arrested in the G2/M phase. Notably, EVs derived from senescent cells contained miR-20a-5p and miR-21-5p, which downregulated the expression of smad7 and promoted the phosphorylation of smad3, thereby activating the TGF-β/Smad pathway. Additionally, these EVs induced macrophages to polarize towards the M2-like phenotype and secrete TGF-β, establishing a positive feedback loop of miR-20a/miR-21-TGF-β. These two mechanisms collectively contributed to the induction of MMT.

To confirm the senescent cell characteristics of impaired renal tubular epithelial cells, we observed a significant increase in the number of renal TECs expressing p21 and p16 in the kidney graft with chronic graft injury and mouse models of renal fibrosis induced by I/R injury, compared to the control group. Furthermore, immunofluorescent staining revealed a substantial increase in p-H3 (ser10)-positive cells in TECs after bilateral I/R injury, indicating cell cycle arrest at the G2/M phase. Notably, the majority of senescent TECs were arrested in the G2/M phase, consistent with previous studies^18^. These senescent TECs in the G2/M phase exhibited elevated levels of pro-fibrotic factors, including TGF-β and CTGF, and activated the JNK signaling pathway. Consequently, it is necessary to establish a reliable method for inducing G2/M phase arrest in HK-2 cells for *in vitro* experiments, although a standardized protocol has not yet been established. In our preliminary experiments, neither hypoxia-reoxygenation conditions nor H_2_O_2_ were able to stably arrest HK-2 cells in the G2/M phase of the cell cycle. Following the experimental protocol of Yang *et al.*^5^, paclitaxel, a microtubule stabilizing agent^20^, was able to irreversibly and stably arrest HK-2 cells in the G2/M phase and similarly exhibit a significant pro-fibrotic phenotype. Therefore, we adopted their protocol, using paclitaxel to induce cellular senescence. Paclitaxel induction was effective in achieving stable G2/M phase arrest, although caution must be exercised when comparing the stimulatory effects of paclitaxel and hypoxic/reoxygenation stimulation on cells.

Regarding the senescent cell-associated secretory phenotype, we detected pro-inflammatory and pro-fibrotic cytokines in the supernatant of senescent HK-2 cells. The concentrations of TNF-α, IL-1β, INF-γ, MCP-1, TGF-β, CTGF, and VEGF were found to increase compared to the control group, with a more prominent elevation observed for pro-fibrotic factors. This observation suggested that senescent renal tubular cells, particularly those arrested in the G2/M phase, predominantly secreted pro-fibrotic factors. Notably, the concentration of TGF-β in the supernatant did not significantly differ from the control group. Conversely, the concentration of CTGF, a crucial pro-fibrotic factor, significantly increased and positively correlated with the proportion of G2/M phase HK-2 cells. CTGF has been implicated in augmenting the binding of TGF-β to its receptors and activating the TGF-β/smad3 pathway^21^-^24^. Furthermore, CTGF exhibited inhibitory effects on BMP7 activity^25^.

In our experiment, we observed that the fibrosis markers of MMT after co-culturing senescent HK-2 cells with THP-1-derived macrophages did not show a clear dose-dependent effect. This may be due to the treatment of HK-2 cells with PTX for 24 hours to induce senescence, followed by co-culturing with macrophages for 48 hours. During co-culturing, senescent HK-2 cells continued to die, and we observed that the rate of HK-2 cell death in the PTX 10μmol/L group was higher compared to the control and PTX 1μmol/L groups. On the other hand, the number of EVs from the PTX-10μmol/L and PTX-1μmol/L groups showed no significant difference. Therefore, we proposed that although the senescent HK-2 cells in the PTX 10μmol/L group may have an increased ability to release EVs, the number of senescent HK-2 cells in this group decreased more rapidly during co-culture compared to the other two groups. As a result, the experimental data did not exhibit the dose-dependent effect that we initially hypothesized.

Our investigation demonstrated that miR-20a-5p and miR-21-5p induced macrophage polarization towards the M2-like subtype in a concentration-dependent manner. Low concentrations of these miRNAs induced M2a polarization, while higher concentrations resulted in M2c polarization. M2 macrophages play a crucial role in anti-inflammatory and injury repair processes by promoting inflammation resolution, tissue remodeling, fibrosis, and wound healing^26^, ^27^. However, the classification of pro-fibrotic macrophages as M2a or M2c remains controversial^28^-^30^. In the study conducted by Luo *et al.*, M2a macrophages facilitate disease progression through macrophage-to-myofibroblast transition, while M2c macrophages exhibit potent anti-inflammatory properties and contribute to tissue repair in renal fibrosis^31^. The intensity of TGF-β signaling is a major determinant of the differential polarization of M2a and M2c macrophages^31^. Furthermore, another study reported that the quantity of CD163+ macrophages at 1- and 5-years post-transplantation was associated with the extent of interstitial fibrosis observed at 5 and 10 years post-transplantation^29^. In our study, we found that macrophages undergoing myofibroblast transformation expressed CD163+M2c markers, and further exploration is warranted to elucidate the underlying regulatory mechanisms. Additionally, miR-21 has been implicated in various processes such as wound healing, angiogenesis, and prevention of tubular cell death^32^-^34^, while miR-20a has been associated with promoting fibrosis in other organs^35^, ^36^. The concentration-dependent effects of miR-21 in the micro-environment may contribute to the repair or fibrosis of injured renal tissues.

In our study, we isolated EVs from senescent HK-2 cells using differential centrifugation, resulting in EVs with a size range of 112-220nm. However, this method did not exclude the presence of cellular debris, potentially leading to contamination with cellular RNA, which could alter the miRNA profiles to some extent^37^. To specifically detect exosomal RNA, RNase A treatment is sometimes applied after exosome isolation from biofluid to degrade any residual cellular RNA^38^. However, RNase A may also degrade miRNAs within the EVs^38^, so we did not use RNase A treatment after centrifugation. As a result, our microRNA sequencing outcomes might deviate from the true miRNA profiles. Additionally, we used snRNA U6 to normalize the expression levels of EV miRNAs in our experiments. U6 snRNA, a nuclear transcript involved in the spliceosome complex of mRNA transcription without post-transcriptional signaling functions^39^, has been recommended for miRNA quantification in pure EVs or vesicle-enriched body fluids^40^. However, it should be acknowledged that the stability of U6 snRNA expression, often used in studies of exosomal miRNA profiling from different biofluids, is not validated^37^. U6 snRNA exhibits high interindividual variability^37^, as well as in whole serum samples from healthy individuals, liver fibrosis patients, and intensive care unit patients^41^. Moreover, while some studies have indicated that U6 snRNA is also present in exosomes, its presence remains controversial and may be attributable to cellular contamination^42^, ^43^.

During the progression of renal fibrosis, TGF-β expression is significantly upregulated, promoting the activation and accumulation of myofibroblasts in the renal interstitium^44^-^46^. Numerous studies have highlighted the critical role of the TGF-β1/SMAD3 signaling pathway in mediating MMT. Smad3 knockout mice exhibit reduced myofibroblast accumulation in various renal fibrosis models^47^-^49^. *In vitro* studies have demonstrated that Src is involved in the TGF-β1/SMAD3 signaling pathway in bone marrow-derived macrophages. Activated SMAD3 binds to the 3′ untranslated region of Src, thereby regulating the MMT process^50^). Tang *et al.* found that the POU domain protein (Pou4f1) functions as a target of SMAD3 and enhances TGF-β1/SMAD3-driven MMT at the transcriptional level, suggesting it may serve as a specific regulator of MMT^51^. Additionally, studies have indicated that the JAK/STAT6 pathway may be an important signaling pathway in the MMT process. In two kidney fibrosis models, the use of JAK or STAT6 inhibitors significantly reduced the number of bone marrow-derived fibroblasts in the renal interstitium^52^-^54^.

Our study revealed that miR-20a-5p and miR-21-5p could suppress the expression of Smad7, which, as a competitive inhibitor, competes with Smad2/3 for binding with the phosphorylated TGF-β type I receptor^55^, ^56^. Thus, miR-20a-5p and miR-21-5p promote the activation of the TGF-β /Smad pathway by inhibiting the expression of Smad7. The phosphorylated Smad2/3 then binds to Smad4, forming a Smad2/3/4 complex that translocates from the cytoplasm into the nucleus, and promotes the expression of pro-fibrotic factors such as α-SMA, FN, and COL1A1^57^. Inhibition of these miRNAs resulted in reduced MMT, particularly evident in the decreased fibronectin protein expression. These findings suggest that macrophage transition is governed by a combination of miRNAs, such as miR-20a and miR-21, and their synergistic interactions, as well as other factors acting in concert.

## Conclusion

In summary, our research provides evidence indicating that during the early stage of ischemia-reperfusion injury, the epithelial cells of renal tubules exhibit cellular senescence. The elimination of senescent cells has the potential to mitigate renal fibrosis and MMT. *In vitro* experiments have demonstrated that senescent HK-2 cells induce MMT by releasing extracellular vesicles. Subsequent analysis has revealed that miR-20a-5p and miR-21-5p present in these vesicles can stimulate macrophages towards M2 polarization and MMT.

## Supplementary Material

Supplementary methods, figures and tables.

## Abbreviations

AKI: Acute kidney injury
IRI: Ischemia-reperfusion injury
CKD: Chronic kidney disease
TEC: Renal tubular epithelial cell
HK-2: Human kidney-2
THP-1: Tohoku Hospital Pediatrics-1
SASP: Senescence-associated secretory phenotype
MMT: Macrophage to myofibroblast transition
α-SMA: α-smooth muscle actin
FN: Fibronectin
miRNA: microRNA
EVs: Extracellular vesicles
NTA: Nanoparticle Tracking Analysis
TEM: Transmission electron microscopy
PTX: Paclitaxel
PMA: Phorbol 12-myristate 13-acetate
INF: Interferon
TGF: Transforming growth factor
VEGF: Vascular endothelial growth factor
KEGG: Kyoto encyclopedia of genes and genomes
mTOR: Mammalian target of rapamycin
