# Supplementary material for: Senescent renal tubular cells derived extracellular vesicles transported miR-20a and miR-21 induced macrophage-to-myofibroblast transition in renal fibrosis after ischemia reperfusion injury
Source: Int J Biol Sci. 2025 Jan 6;21(3):940–54. doi: 10.7150/ijbs.97579 (PMC11781180; doi:10.7150/ijbs.97579)
Supplement: Supplementary file 1 — Supplementary methods, figures and tables. [file ijbsv21p0940s1.pdf]

Supplementary Table 2 Patient Demographics

Characteristics	Case 1	Case 2
Age, yr	49	34
Gender	male	male
Timing of biopsy post-transplantation, mo	98	63
Type of kidney transplantation	Living donor	Living donor
No. of HLA mismatch	4	4
Type of immunosuppressive induction	ATG	ATG
Immunosuppressive maintenance	Tac+MMF+steroid	Tac+MMF+steroid
Hypertension at time of biopsy	Yes	Yes
Proteinuria at time of biopsy	1.8g/24h	2.1g/24h
Serum creatinine at time of biopsy	186μmol/L	157μmol/L
Banff scores (2019)	i0, t0, g1, v0, ci1, ct1, cg1b, cv1, ahh3, mm2, ptc0, ti0, i-IFTA0, t-IFTA0, C4d+	i1,t0,g1,v0,ci1,ct1,cg2,cv1,ahh3,mm2,ptc1,g+ptc=2,ti1,i-IFTA1,t-IFTA0,C4d-

Tac, tacrolimus; MMF, mycophenolate mofetil; i, interstitial inflammation; t, tubulitis; v, intimal arteritis; g, glomerulitis; ci, interstitial fibrosis; ct, tubular atrophy; cv, vascular fibrous intimal thickening; cg, allograft glomerulopathy; ptc, glomeruli and peritubular capillaries; mm, mesangial matrix expansion; ahh, hyaline arteriolar thickening; ti, total inflammation; i-IFTA, Inflammation in area of IFTA; t-IFTA, tubulitis in areas of interstitial fibrosis.

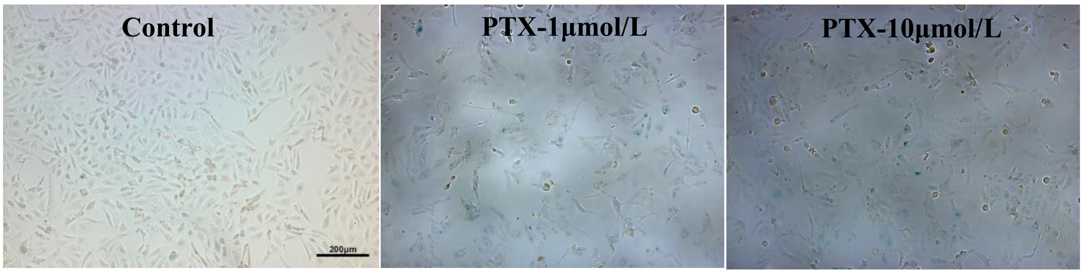

Supplementary Figure 1. Cell senescence was measured by SA-β-gal staining. The cells were stained for the presence of senescence-associated SA β-galactosidase activity.

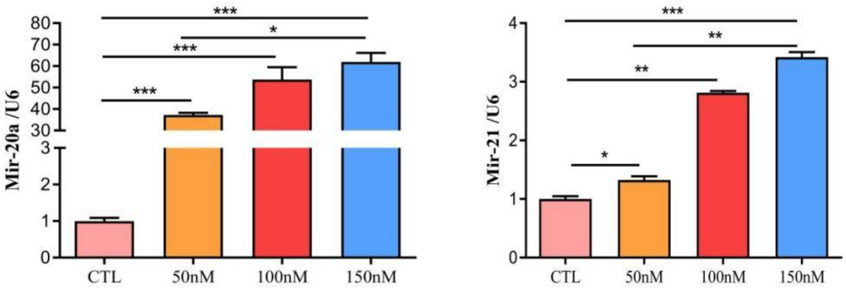

Supplementary Figure 2 The significantly high-expressed miR-20a-5p or miR-21-5p in macrophage after transfection of the respective mimic. The relative expression of miR-20a-5p or miR-21-5p in macrophages increased in a concentration-dependent manner with the increase of the concentration of the mimics.***P<0.001;**P<0.01;*P<0.05

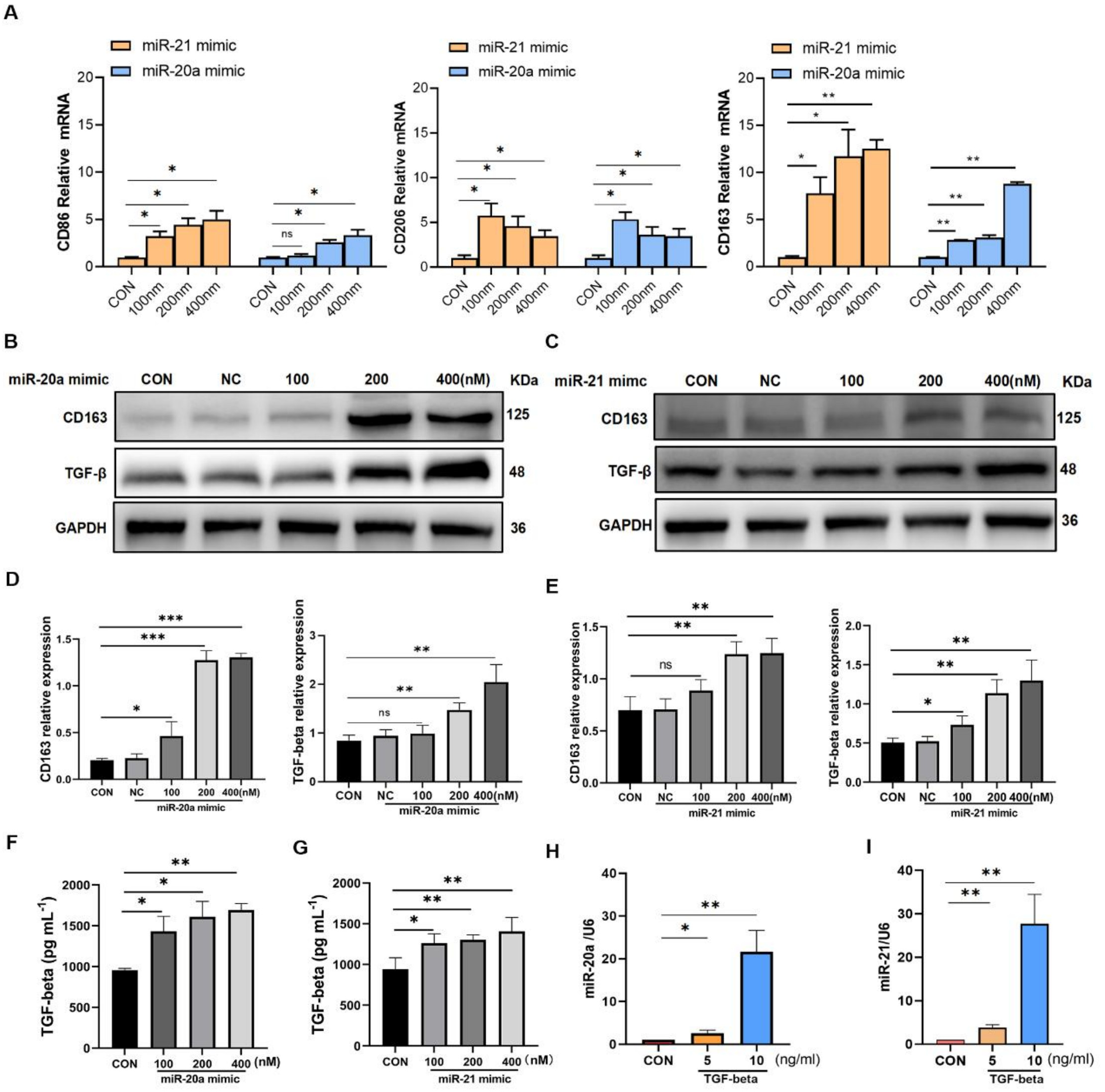

Supplementary Figure 3. MiR-20a-5p and miR-21-5p promoted macrophage M2-like polarization and formed a positive feedback loop with TGF-β. (A) Macrophage were transfected with miR-20-5p mimic or miR-21-5p mimic for 48 hours, after which CD86, CD206 and CD163 mRNA relative expression were assayed by qPCR. (B) Macrophage were transfected with miR-20a-5p mimic or control miR for 48 hours, after which CD163 and TGF-β protein expression were assayed by western blot. (C) Macrophage were transfected with miR-21a-5p mimic or control miR for 48 hours, after which CD163 and TGF-β protein expression were assayed by western blot. (D) Quantified data of immunoblotting band intensity in (B). (E) Quantified data of immunoblotting band intensity in (C). (F) Macrophage were transfected with miR-20a-5p mimic for 48 hours, after which the concentrations of TGF-β in supernatants were assayed by ELISA. (G) Macrophage were transfected with miR-21-5p mimic for 48h, after which the concentrations of TGF-β in supernatants were assayed by ELISA. (H) MiR-20a-5p relative expression were detected by qPCR in macrophage treated with TGF-β (5ng/ml,10ng/ml) for 48h. (I) MiR-21-5p relative expression were detected by qPCR in macrophage treated with TGF-β(5,10ng/ml) for 48h. ***P<0.001; **P<0.01; *P<0.05; ns, not significant.

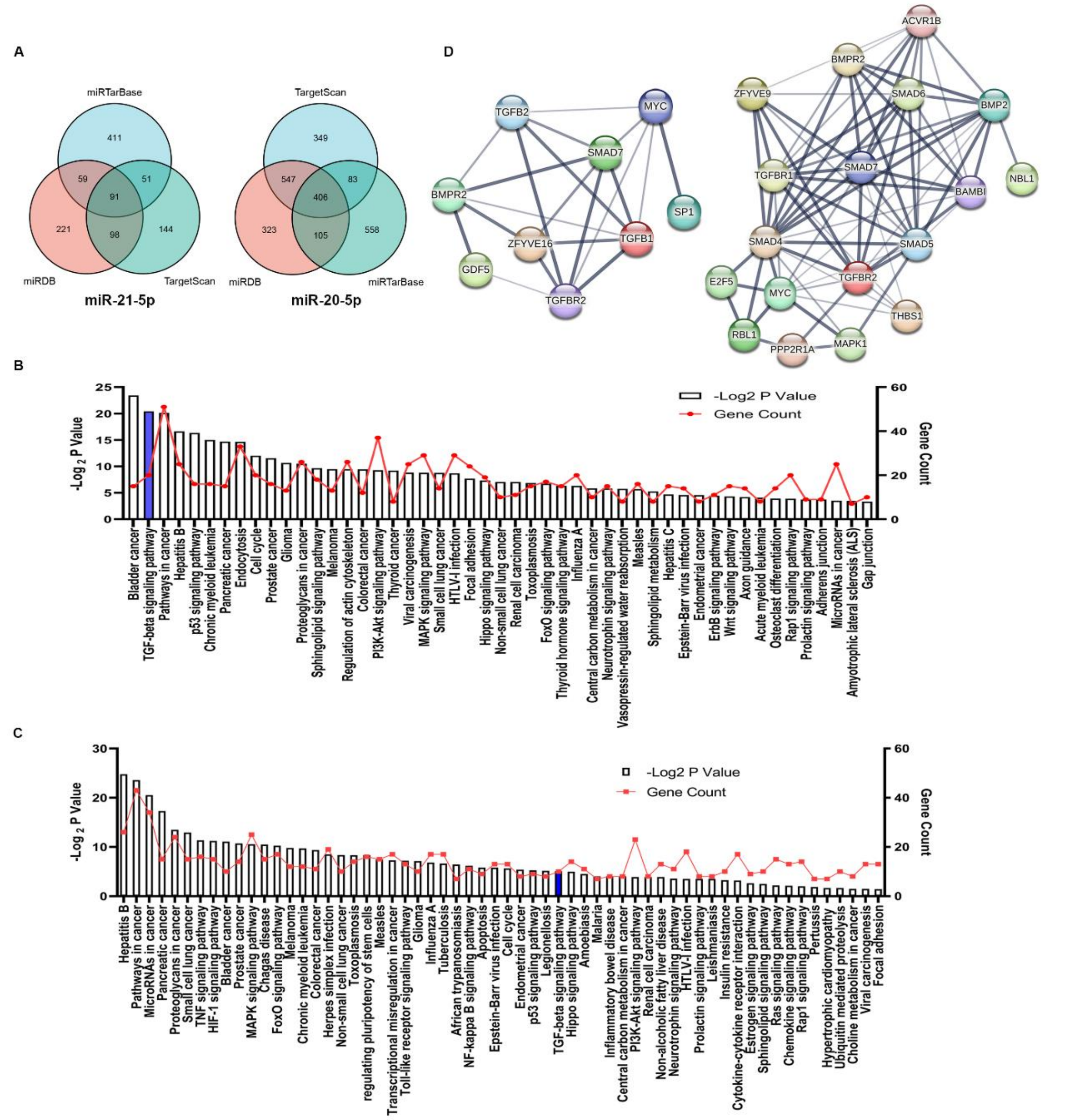

Supplementary Figure 4. Identification of the signaling pathway and the activated proteins. (A) Venn diagram of target genes predicted by miRDB, TargetScan, and miRtarbase. (B) KEGG pathway enrichment analysis of target genes of miR-20a-5p. The red lines represent gene count and the histogram represent-log2 (P value). (C) KEGG pathway enrichment analysis of target genes of miR-21-5p. The red lines represent gene count and the histogram represent-log2 (P value). (D) Analysis of protein-protein interaction networks within the TGF-β signaling pathway (left, miR-21-5p targeted gene; right, miR-20-5p targeted gene); the networks created using String software version 11.0 with minimal confidence score of 0.4, and the thickness of the line indicates the strength of the interaction based on the supporting data.

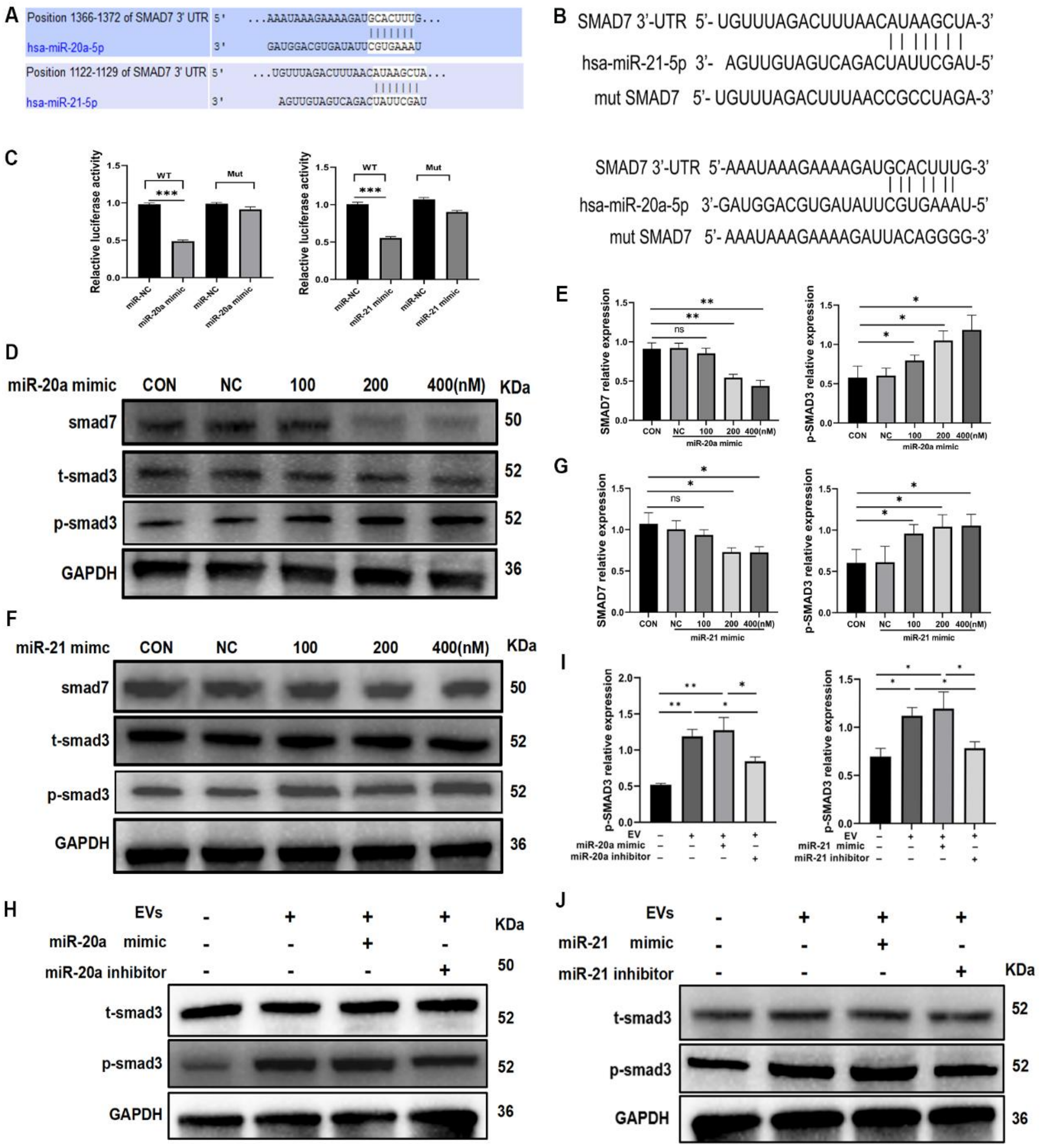

Supplementary Figure 5. MiR-20a-5p and miR-21-5p inhibited SMAD7 expression and promoted SMAD3 phosphorylation. (A) Predicted binding site between miR-20a-5p/miR-21-5p and SMAD7 using TargetScan software. (B, C) Dual luciferase reporter assays demonstrated that miR-20a-5p and miR-21-5p could bind to the 3'UTR of SMAD7 mRNA. (D, F) MiR-20a-5p or miR-21-5p overexpression in macrophages suppress Smad7 and activate p-Smad3 protein expression as assessed by western blot. (E, G) Quantified data of immunoblotting band intensity in (D, F). (H, J) Western blotting confirms that miR-20a-5p or miR-21-5p mimic further enhances sHK-2-EVs induced p-Smad3 activation, and miR-20a-5p or miR-21-5p inhibitor counteracts the elevation of p-Smad3 protein induced by sHK-2-EVs in macrophages. (I) Quantified data of immunoblotting band intensity in (H, J). **P<0.01; *P<0.05; ns, not significant.

Supplementary Methods

1. Transwell

For the transwell assay, PMA-pretreated macrophages and PTX-pretreated HK-2 cells were placed in the bottom and top chambers, respectively, of a transwell chamber system (pore size 0.4μm, Corning, USA). After 48 hours of co-culture, the macrophages were harvested for subsequent experiments.

2. EVs isolation

Initially, the cell supernatant was centrifuged at 300g for 5 minutes to eliminate dead cells and cellular debris. Next, the supernatant was subjected to centrifugation at 2000g for 10 minutes, followed by an additional centrifugation step at 10,000g for 10 minutes at 4°C. Subsequently, the resulting supernatant was centrifuged at 120,000g for 80 minutes at 4°C using an Optima XPN Ultracentrifuge (Beckman Coulter, CA, USA) to collect the EVs. The EVs were then washed once with precooled PBS and resuspended in 100μL of precooled PBS. Throughout the isolation process, the cell supernatant obtained after EVs isolation was preserved for subsequent experiments. Then EVs were confirmed by Transmission electron microscopy and Nanoparticle tracking analysis.

3. Transmission electron microscopy

The isolated extracellular vesicles (EVs) derived from senescent HK-2 cells were subjected to fixation using 2.5% glutaraldehyde stationary liquid. Subsequently, EV suspensions were applied onto carbon-coated formvar grids for 5 minutes and stained with 2% phosphotungstic acid at room temperature for an additional 5 minutes. A collection of EV images was captured using a JEM-1400Flash transmission electron microscope (JEOL, Tokyo, Japan) operating at 80 kV.

4. Nanoparticle tracking analysis (NTA)

The concentration and particle size of the extracellular vesicles (EVs) were assessed using the ZetaView Z-NTA system (Particle Metrix, Meerbusch, Germany) along with the corresponding software (ZetaView 8.04.02).

5. ELISA

The concentrations of SASP, including TNF-α, IL-1β, INF-γ, CTGF, MCP-1, VEGF, and TGF-β in the supernatant of senescent HK-2 cells were quantified using ELISA kits obtained from Ruixinbio, following the manufacturer's instructions. Similarly, the TGF-β levels in the macrophage cell supernatant were determined using ELISA kits from eBioscience, following the manufacturer's guidelines.

6. SA-β-gal assay

The cellular senescence of HK-2 cells was evaluated using a SA-β-gal staining kit (Beyotime, C0602). In brief, HK-2 cells were seeded in 6-well plates and treated with various concentrations of paclitaxel for 24 hours. Following three washes with PBS, the HK-2 cells were fixed for 20 minutes and subsequently incubated with the SA-β-gal staining solution at 37°C overnight. Finally, the SA-β-gal positive cells were visualized as blue-stained cells.

7. Cell cycle assay

Following a 24-hour treatment with various concentrations of paclitaxel, the HK-2 cells were detached using trypsin-free EDTA and harvested through centrifugation. After resuspending the cells in PBS, the suspensions were fixed overnight at 4°C in ice-cold 70% ethanol. Subsequently, the cells were centrifuged at 800 rpm for 5 minutes, resuspended in 50 μl of RNase A, and incubated at 37°C for 30 minutes. Following this, 400 μl of propidium iodide was added to the suspension and incubated for an additional 30 minutes. The cell samples were then analyzed using flow cytometry (BD Biosciences, CA, USA).

8. PKH67 staining for EVs and internalization

To label the lipid bilayers of EVs, PKH67 Green Fluorescent Cell Linker Kits (NoninBio, Shanghai, China) were utilized. In brief, approximately 10^{10}/ml EVs were resuspended in 25 μl of PBS. Then, the dye working solution (50 μl) was added to the EVs suspension, followed by mixing on a vortex shaker for 1 minute. The mixture was allowed to rest and incubate for 10 minutes. Subsequently, 10 ml of PBS was added to the incubated EVs-dye complex, and pure PKH67-labeled EVs were obtained through differential centrifugation. The labeled EVs were then

resuspended in complete medium and incubated with THP-1-derived macrophages at 37°C. After 48 hours, the cells were washed, fixed for 30 minutes, and then stained with DAPI for an additional 15 minutes. for 24 hours. To visualize the incorporation of EVs into the macrophages, immunofluorescence microscopy was performed using an Olympus IX-71 microscope (Olympus, Tokyo, Japan).

9. H&E, Masson's trichrome and Immunohistochemical (IHC) staining

The primary antibodies used for HE and IHC staining included monoclonal rabbit anti-human p21 (1:100, ab109520, Abcam), monoclonal rabbit anti-mouse p21 (1:800, ab188224, Abcam), monoclonal rabbit anti-mouse F4/80 (1:200, #70076, CST), monoclonal rabbit anti-mouse COL1A1 (1:100, #72026, CST), and monoclonal rabbit anti-mouse α-SMA (1:50, ab5694, Abcam).

10. Immunofluorescence staining

The primary antibodies for immunofluorescence staining included monoclonal rabbit anti-human fibronectin (FN) (1:200, ab45688, Abcam), monoclonal rabbit anti-human COL1A1 (1:100, #72026, CST), and monoclonal rabbit anti-mouse α-SMA (1:250, ab124964, Abcam). Fluorescent secondary antibodies, namely goat anti-rabbit IgG-Alexa Fluor® 488 (1:500, ab150081) and goat anti-rabbit IgG-Alexa Fluor® 647 (1:500, ab150079), were obtained from Abcam. Nuclei were stained using DAPI (#4083, CST). The samples were visualized using immunofluorescence microscopy (Olympus, Tokyo, Japan). For fluorescent multiplex immunohistochemistry, OPAL Multiplex Fluorescent Immunohistochemistry Reagents (PerkinElmer, Waltham, MA, USA) were employed following the manufacturer's protocol. Formalin-fixed paraffin-embedded sections were stained using one of the three sequences of primary antibodies. The primary antibody sequences used were: F4/80 (1:200, #30325, CST) or CD68 (1:200, #76437, CST), COL1A1 (1:200, ab34710, Abcam) and α-SMA (1:300, ab7817, Abcam); or F4/80 (1:200, #30325, CST), p21 (1:1000, ab1888275, Abcam), and p-H3(ser10) (1:100, #53348, CST).

11. Western blot analysis

The primary antibodies used included p53 (1:1000, #2524, CST), p21 (1:1000, ab109520, Abcam), p16 (1:1000, #80772, CST), GAPDH (1:10000, ab181602, Abcam), p-γ-H2AX (Ser139) (1:800, #9718, CST), FN (1:1000, #A12932, Abclonal), α-SMA (1:1000, #A17910, Abclonal), COL1A1 (1:500, #A1352, Abclonal), TSG101 (1:1000, ab125011, Abcam), CD81 (1:500, ab79559, Abcam), CD63 (1:1000, ab134045, Abcam), β-Tubulin (1:1000, #2146, CST), Smad3 (1:1000, #AF6362, Affinity), p-Smad3 (Ser423+Ser425) (1:1000, #AF8315, Affinity), CD163 (1:2000,

#DF8235, Affinity), and Smad7 (1:1000, #AF5147, Affinity). On the following day, the membranes were washed and incubated with horseradish peroxidase (HRP)-conjugated secondary antibodies: Goat Anti-Rabbit IgG (H+L) (1:5000, #AS014, Abclonal) and Goat Anti-Mouse IgG (H+L) (1:5000, #AS003, Abclonal). The proteins were visualized using an enhanced chemiluminescence detection reagent (Affinity) and quantified using ImageJ software (version 2.0.0, ImageJ, Marlyand, USA).

12. Supplementary Table 1 List of primers for qPCR

Primers	Sequence (5' to 3')
CD86 Forward	CTGCTCATCTATACACGGTTACC
CD86 Reverse	GGAAACGTCGTACAGTTCTGTG
CD206 Forward	GGGTTGCTATCACTCTCTATGC
CD206 Reverse	TTTCTTGTCTGTTGCCGTAGTT
CD163 Forward	TTGTCAACTTGAGTCCCTTCAC
CD163 Reverse	TCCCGCTACACTTGTTTTCAC
TGFB1 Forward	CTGTACATTGACTTCCGCAAG
TGFB1 Reverse	TGTCCAGGCTCCAAATGTAG
GAPDH Forward	ACAACTTTGGTATCGTGGAAGG
GAPDH Reverse	GCCATCACGCCACAGTTTC
hsa-U6 Forward	CTCGCTTCGGCAGCACA
hsa-U6 Reverse	AACGCTTCACGAATTTGCGT
hsa-miR-20a-5p Forward	GCGCGTAAAGTGCTTATAGTGC
hsa-miR-20a-5p Reverse	AGTGCAGGGTCCGAGGTATT
hsa-miR-21-5p Forward	GCGCGTAGCTTATCAGACTGA
hsa-miR-21-5p Reverse	AGTGCAGGGTCCGAGGTATT
hsa-miR-182-5p Forward	GCGTTTGGCAATGGTAGAACT
hsa-miR-182-5p Reverse	AGTGCAGGGTCCGAGGTATT
hsa-miR-16-5p Forward	CGCGTAGCAGCACGTAAATA
hsa-miR-16-5p Reverse	AGTGCAGGGTCCGAGGTATT
miRNA primer (stem-loop)	GTCGTATCCAGTGCAGGGTCCGAGGTATTCGCACTGGATACGACC GCCAA

13.The letter of ethics approval

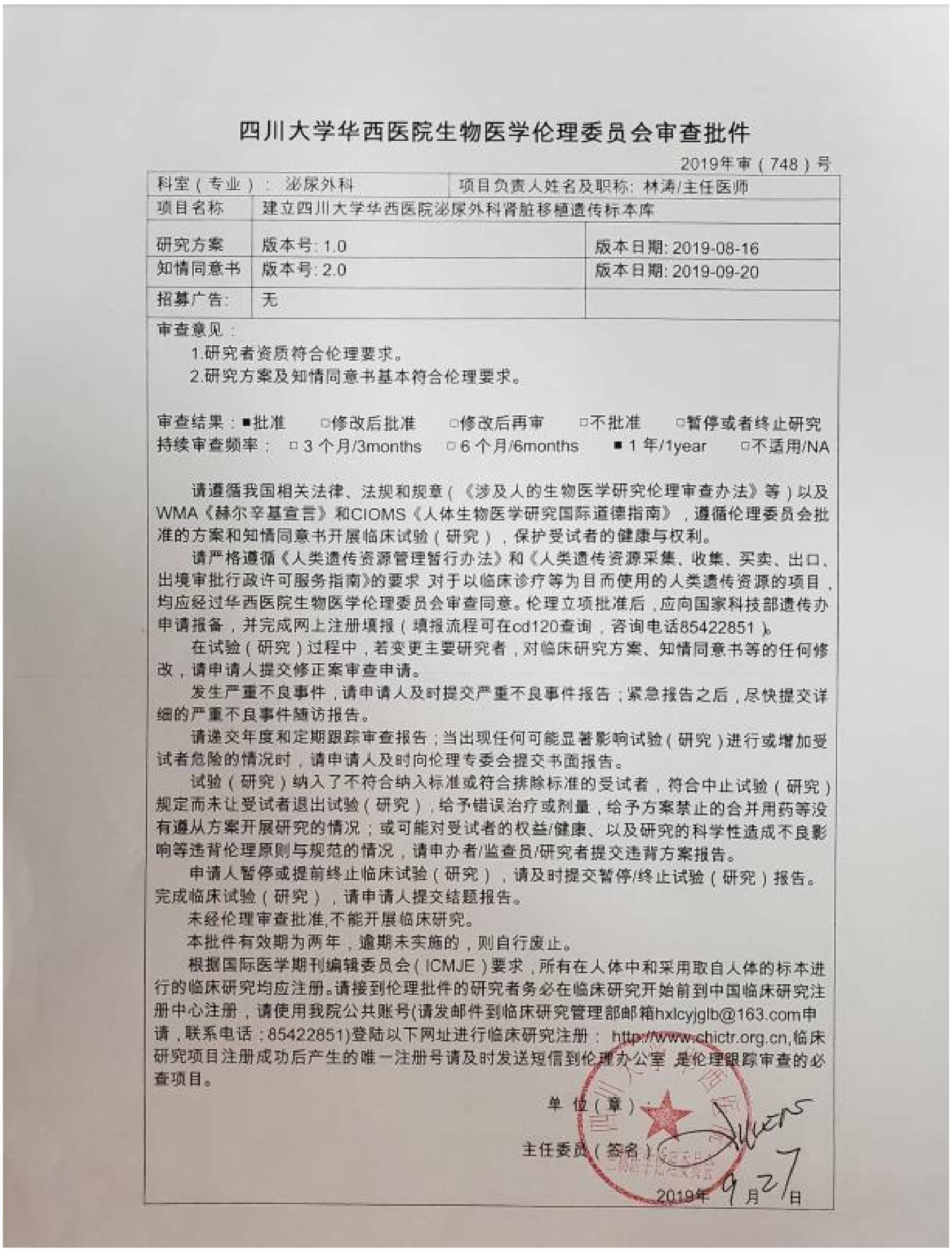

四川大学华西医院生物医学伦理委员会审查批件

2019年审（748）号

科室（专业）：泌尿外科	项目负责人姓名及职称：林涛/主任医师	
项目名称	建立四川大学华西医院泌尿外科肾脏移植遗传标本库	
研究方案	版本号：1.0	版本日期：2019-08-16
知情同意书	版本号：2.0	版本日期：2019-09-20
招募广告：	无	

审查意见：

1.研究者资质符合伦理要求。

2.研究方案及知情同意书基本符合伦理要求。

审查结果：■批准　□修改后批准　□修改后再审　□不批准　□暂停或者终止研究

持续审查频率：□ 3 个月/3months　□ 6 个月/6months　■ 1 年/1year　□不适用/NA

请遵循我国相关法律、法规和规章（《涉及人的生物医学研究伦理审查办法》等）以及WMA《赫尔辛基宣言》和CIOMS《人体生物医学研究国际道德指南》，遵循伦理委员会批准的方案和知情同意书开展临床试验（研究），保护受试者的健康与权利。

请严格遵循《人类遗传资源管理暂行办法》和《人类遗传资源采集、收集、买卖、出口、出境审批行政许可服务指南》的要求 对于以临床诊疗等为目而使用的人类遗传资源的项目，均应经过华西医院生物医学伦理委员会审查同意。伦理立项批准后，应向国家科技部遗传办申请报备，并完成网上注册填报（填报流程可在cd120查询，咨询电话85422851）。

在试验（研究）过程中，若变更主要研究者，对临床研究方案、知情同意书等的任何修改，请申请人提交修正案审查申请。

发生严重不良事件，请申请人及时提交严重不良事件报告；紧急报告之后，尽快提交详细的严重不良事件随访报告。

请递交年度和定期跟踪审查报告；当出现任何可能显著影响试验（研究）进行或增加受试者危险的情况时，请申请人及时向伦理专委会提交书面报告。

试验（研究）纳入了不符合纳入标准或符合排除标准的受试者，符合中止试验（研究）规定而未让受试者退出试验（研究），给予错误治疗或剂量，给予方案禁止的合并用药等没有遵从方案开展研究的情况；或可能对受试者的权益/健康、以及研究的科学性造成不良影响等违背伦理原则与规范的情况，请申办者/监查员/研究者提交违背方案报告。

申请人暂停或提前终止临床试验（研究），请及时提交暂停/终止试验（研究）报告。完成临床试验（研究），请申请人提交结题报告。

未经伦理审查批准,不能开展临床研究。

本批件有效期为两年，逾期未实施的，则自行废止。

根据国际医学期刊编辑委员会（ICMJE）要求，所有在人体中和采用取自人体的标本进行的临床研究均应注册。请接到伦理批件的研究者务必在临床研究开始前到中国临床研究注册中心注册，请使用我院公共账号(请发邮件到临床研究管理部邮箱hxlcyjglb@163.com申请，联系电话：85422851)登陆以下网址进行临床研究注册：http://www.chictr.org.cn,临床研究项目注册成功后产生的唯一注册号请及时发送短信到伦理办公室 是伦理跟踪审查的必查项目。

单位（章）：

主任委员（签名）：

2019年 9 月 27 日
